# Editorial: Modulating cytokines as treatment for autoimmune diseases and cancer: volume II

**DOI:** 10.3389/fimmu.2023.1225699

**Published:** 2023-06-05

**Authors:** Gaby Palmer, Sheela Ramanathan, Erwan Mortier

**Affiliations:** ^1^ Division of Rheumatology, Department of Medicine, Faculty of Medicine, University of Geneva, Geneva, Switzerland; ^2^ Department of Pathology and Immunology, Faculty of Medicine, University of Geneva, Geneva, Switzerland; ^3^ Geneva Centre for Inflammation Research, Faculty of Medicine, University of Geneva, Geneva, Switzerland; ^4^ Department of Immunology and Cell Biology, Faculty of Medicine and Health Sciences, Université de Sherbrooke, Sherbrooke, QC, Canada; ^5^ Nantes Université, CNRS, Inserm, CRCI2NA, Nantes, France; ^6^ LabEX IGO, Immuno-Onco-Greffe, Nantes, France

**Keywords:** cytokine, oncology, inflammation, inhibitor, therapy, leukemia, lymphoma

## Introduction

Cytokines play a crucial role in the regulation of immune responses. Dysregulated cytokine production causes various pathologies, including autoimmunity and cancer. Understanding the different modes of action of cytokines is of major interest, especially for the design of new selective drugs that modulate their activities and to exploit their prognostic value.

In the second volume of the Research Topic “*Modulating cytokines as treatment of autoimmune diseases and cancer*”, we have compiled 5 original research articles and 2 reviews. This collection is divided into four sections, respectively discussing the role of cytokines in epithelia-derived tumors, selective targeting of IL-15 in cancer and inflammation, the role played by cytokines in hematological malignancies and the therapeutic interest of an IL-22 derived immunocytokine.

## Role of cytokines in epithelia-derived solid tumors

In barrier tissues, epithelial cell-derived cytokines play an important role in orchestrating innate and adaptive immune responses against invading pathogens. Epithelia are also the most common site for the development of cancers. In this context, epithelial cell-derived cytokines can profoundly affect cancer development and exert pro- or anti-tumor activities through their effects on immune cells.

Colorectal cancer (CRC) develops in the complex environment of the gut, where cytokines play an important role in maintaining barrier integrity and host-microbe symbiosis. Epithelial cell-derived IL-25 and IL-33 are potent activators of type 2 immune responses, and promote intestinal tissue repair and homeostasis (1, 2). In the last decade, a role for IL-25 and IL-33 has also emerged in the development of CRC. Jou et al. give an excellent overview of our current knowledge regarding overlapping and distinct functions of IL-25 and IL-33 in CRC and the prospects for immunotherapy targeting these cytokines.

Cutaneous melanoma is an immunogenic tumor, but suppressive mechanisms limit the anti-tumor immune response. Melanoma-associated mast cells have been implicated in immunotherapy resistance (3), although the mechanisms by which mast cells influence melanoma development are unclear. Bahri et al. examined the effects of the malignant melanoma microenvironment on tumor-associated mast cells. Their study emphasizes the role of mediators secreted by melanoma cells, and in particular cytokines, in promoting a mast cell phenotype that is characterized by expression of the complement component C3 and associated with poor prognosis.

Together, these two contributions illustrate the prominent role of cytokines in the complex crosstalk between tumor cells and the immune system in epithelial cancers. Interestingly, while current immunotherapies focus primarily on adaptive anti-tumor immunity and T cells, these two articles highlight actors of the innate immune system, supporting the emerging notion that targeting innate immune signals may represent an attractive complementary approach to existing therapies for solid cancers.

## Fine-tuning of IL-15 action in cancer and inflammation

Despite sharing the two signaling subunits IL-2Rβ (CD122) and γ_c_ (CD132) with IL-2, IL-15 has distinct functions. IL-15 activates NK cells and is required for the maintenance of memory CD8^+^ T cells and other lymphocytes. The bioactivity of IL-15 on CD8^+^ T and NK cells relies on its ‘*trans-*presentation’ by producer cells where the ‘IL-15:IL-15Rα’ complex is presented to IL-2Rβ/γ_c_ on responder cells, as opposed to ‘*cis-*presentation’ where IL-15 signals through its receptors on the responder cell. IL-15 mediated activation of NK cells and CD8^+^ T cells, that are dependent on IL-15 *trans*-presentation, are important in anti-tumor immune responses (4). However, use of native IL-15 in immunotherapies induces cytokine storm (5). Alternative approaches include covalent complexing of IL-15 with the interacting domain of IL-15Rα (6, 7). SOT101 (formerly RLI-15) is a monomeric soluble construct of IL-15 coupled covalently with the IL-15Rα sushi domain. It is in phase 1 and 2 clinical trials as a monotherapy or in combination with other immune checkpoint inhibitors. Antosova et al. analyzed the functionality of NK cells activated by SOT101 in promoting ADCC induced by Cetuximab, Daratumumab and Obinutuzumab, that target epidermal growth factor receptor, CD38 and CD20 respectively.

In autoimmune diseases, the mechanism of action and cell types targeted by IL-15 are not very clear (8). Studies from pre-clinical models suggest that IL-15 may activate the inflammatory processes independent of NK cells and other lymphocyte subsets. An antibody directed against IL-15 is in phase 1 clinical trial in refractory celiac disease (9) and showed promising results. Meghnem et al., show that NANTIL-15 (for New ANTagonist of IL-15, from Nantes, pun intended) was effective in a pre-clinical model of collagen-induced arthritis. Mutations introduced in IL-15 prevent its interaction with the IL-2Rβ subunit, but not with IL-15Rα. Thus, NANTIL-15 acts as a competitive inhibitor for the trimeric receptor, without affecting *trans*-presentation of endogenous IL-15. It can be hoped that, in future, NANTIL-15 will help to understand the role of IL-15 in inflammation associated with autoimmunity.

## Role of cytokines in hematological cancers

Cytokines play an important role in the development and differentiation of immune cells. Under certain conditions, dysregulation of cytokine expression occurs, leading to hematological malignancies. Sindaco et al. reviewed the involvement of IL-15 overexpression in subtypes of leukemia and lymphoma, and other hematologic diseases and provide an overview of IL-15-based therapeutic approaches to counteract the deleterious action of IL-15 in these diseases.

Despite recent advances in the management of acute promyelocytic leukemia (APL) the early death (ED) rate remains high. Zhao et al. found that APL occurs preferentially in younger patients and is associated with a high inflammatory state. Cytokine levels were higher in APL than in other types of acute myeloid leukemia. Elevated levels of IL-17A and TNF-β were directly related to ED in newly diagnosed APL patients, and IL-17A was associated with intracranial hemorrhage, a major contributor to ED. These observations suggest that IL-17A is a predictor of ED in patients with APL.

## Therapeutic use of an IL-22 derived fusion protein

Diabetic nephropathy (DN) is a common and serious complication of diabetes mellitus (10). DN may be associated with podocyte damage, abnormalities in glucose and lipid metabolism, and chronic inflammation. Ma et al. designed a bifunctional immunocytokine, in which an anti-ANGPTL3 blocking antibody is fused to IL-22, a cytokine described to suppress inflammation. In a mouse model of DN, the anti-ANGPTL3/IL22 fusion protein was more effective in reducing proteinuria and improving glucolipid metabolism than either IL-22-Fc or anti-ANGPTL3, or the combination of both molecules. The treatment also significantly attenuated renal fibrosis, suggesting that it may represent a promising new therapeutic strategy for DN.

Collectively, the above studies confirm the key role of cytokines in pathogenesis. Increased knowledge of cytokine biology opens new avenues for the design of therapeutic options aimed at reprogramming cytokine responses in disease.

## Author contributions

All authors listed have made a substantial, direct, and intellectual contribution to the work and approved it for publication.
